# Loss of nonsense mediated decay suppresses mutations in *Saccharomyces cerevisiae TRA1*

**DOI:** 10.1186/1471-2156-13-19

**Published:** 2012-03-22

**Authors:** Stephanie Kvas, Gregory B Gloor, Christopher J Brandl

**Affiliations:** 1 Department of Biochemistry, Schulich School of Medicine & Dentistry, The University of Western Ontario, London N6A5C1, Canada

**Keywords:** Tra1, Yeast, Nonsense mediated decay, Upf1, Gene expression, Second-site suppression

## Abstract

**Background:**

Tra1 is an essential protein in *Saccharomyces cerevisiae*. It was first identified in the SAGA and NuA4 complexes, both with functions in multiple aspects of gene regulation and DNA repair, and recently found in the ASTRA complex. Tra1 belongs to the PIKK family of proteins with a C-terminal PI3K domain followed by a FATC domain. Previously we found that mutation of leucine to alanine at position 3733 in the FATC domain of Tra1 (*tra1-L3733A*) results in transcriptional changes and slow growth under conditions of stress. To further define the regulatory interactions of Tra1 we isolated extragenic suppressors of the *tra1-L3733A *allele.

**Results:**

We screened for suppressors of the ethanol sensitivity caused by *tra1-L3733A*. Eleven extragenic recessive mutations, belonging to three complementation groups, were identified that partially suppressed a subset of the phenotypes caused by tra*1-L3733A*. Using whole genome sequencing we identified one of the mutations as an opal mutation at tryptophan 165 of *UPF1/NAM7*. Partial suppression of the transcriptional defect resulting from *tra1-L3733A *was observed at *GAL10*, but not at *PHO5*. Suppression was due to loss of nonsense mediated decay (NMD) since deletion of any one of the three NMD surveillance components (*upf1/nam7, upf2/nmd2*, or *upf3*) mediated the effect. Deletion of *upf1 *suppressed a second FATC domain mutation, *tra1-F3744A*, as well as a mutation to the PIK3 domain. In contrast, deletions of SAGA or NuA4 components were not suppressed.

**Conclusions:**

We have demonstrated a genetic interaction between *TRA1 *and genes of the NMD pathway. The suppression is specific for mutations in *TRA1*. Since NMD and Tra1 generally act reciprocally to control gene expression, and the FATC domain mutations do not directly affect NMD, we suggest that suppression occurs as the result of overlap and/or crosstalk in these two broad regulatory networks.

## Background

Tra1 is a 3744 amino acid residue protein, essential for viability in *Saccharomyces cerevisiae*. It is a major constituent of the SAGA and NuA4 transcriptional regulatory complexes [1-3], both with significant roles in gene regulation and DNA repair [4-6]. More recently a putative complex based on mutual associations termed ASTRA, was also found to contain Tra1 [7]. Tra1's mammalian homolog TRRAP was identified because of its interactions with the transcription factors c-myc and E2F [8]. Similarly Tra1 interacts with yeast transcriptional activators to target SAGA and NuA4 to promoters [9-12]. Interestingly, Helmlinger et al. [13] have recently provided evidence that Tra1 also acts independently of SAGA and NuA4 to regulate gene expression.

Tra1/TRRAP are members of the PIKK (phosphoinositide three-kinase-related kinase) family of proteins, a group that in yeast includes Tor1, Tor2, Tel1 and Mec1 [14,15]. The latter two are structurally and functionally related to ATM and ATR of multicellular eucaryotes. Two additional family members not found in yeast, are the DNA-PKcs (DNA-dependent protein kinase catalytic subunit) and SMG-1, with key roles in DNA repair and nonsense mediated decay of RNA, respectively. The PIKK family members are all large proteins characterized by a common arrangement of C-terminal domains [16], including a domain that resembles the phosphatidylinositol-3-kinases (PI3K). Unlike the other PIKK molecules, which are protein kinases, Tra1/TRRAP lacks kinase activity [2,8]. Nonetheless, altering residues that parallel key regions of the kinase members of the family affect Tra1 function [17]. On the N-terminal side of the kinase domain is the HEAT and TPR repeat-rich FAT (FRAP-ATM-TRRAP) domain [18-21]. C-terminal to the PI3K domain is the less highly conserved PRD (PIKK regulatory domain), identified in ATM as the site of acetylation by TIP60 [22].

The ~35-residue FATC domain is at the extreme C-terminus of the PIKK proteins [18]. The critical role of the FATC domain is evident from the finding that addition of a single glycine to the C-terminus of Tra1 abolishes function, and mutations of L3733 or F3744 to alanine result in slow growth in a number of stress conditions [23]. The FATC domain is similarly important for the other PIKK family members; for example, the parallel mutation to L3733A of Tra1 results in a dramatic loss in the kinase activity of SMG-1 [24]. Dames et al. [25] determined the structure of the isolated FATC domain of *S. cerevisiae *Tor1. It is predominately helical with a loop at the extreme C-terminus held in place by a disulphide linkage. The helical structure is likely conserved in the family, but not the loop because the two cysteines are only found in the Tor proteins. FATC domains are proposed to be a target for interacting proteins. In a two-hybrid analysis the FATC domain of Mec1 was required for association with the RPA components Rfa1 and Rfa2 [26]. *In vivo*, the FATC domain of ATM and Tip60 (the mammalian homolog of the NuA4 component Esa1) interact, though this may be indirect [22,27]. Consistent with the mutagenesis analysis of Moritia et al. [24], Lempiäinen and Halazonetis [16] suggest the FATC domain interacts with and regulates the activity of the kinase domain.

Nonsense mediated decay (NMD) is a cellular surveillance mechanism present in all eucaryotes that scans for premature stop codons on mRNAs [28,29]. The process is coupled to translation, and results in degradation of potentially deleterious transcripts [30-32]. Premature stop codons can arise through errors in transcription by RNA Polymerase II, failure or inaccurate removal of introns, inaccurate translational starts, ribosomal frameshifting, RNA editing, or errors within the genomic DNA. The mechanism of NMD varies in different organisms, with the molecular details still being determined [33]. In yeast, one model suggests that NMD is triggered if the mRNA binding protein Hrp1 is bound to a downstream sequence element(s) (DSE) when the ribosome encounters a stop codon [34-36]. The DSE is 5' of the native stop codon, with Hrp1 normally being displaced by the passing ribosome. A second model, the faux (false) 3' UTR model, postulates that the length of the 3' untranslated region resulting from a premature stop codon prevents the normal interactions that occur between the ribosome and poly (A) binding protein during the termination of translation. In the absence of these interactions the NMD factors associate with the ribosome, which in turn results in mRNA decay [37].

The yeast NMD surveillance complex consists of three proteins, Upf1/Nam7, Upf2/Nmd2, and Upf3 (for simplicity referred to as Upf1, Upf2 and Upf3, respectively). Mutations in any of the NMD proteins increase translational read-through and stabilize mRNA containing premature stop codons [30,38-43]. Upf1, an ATP dependent RNA helicase [41,44] is the central component of the pathway, whereas Upf2 and Upf3 regulate Upf1 [42,45]. In metazoans, Upf1 is further regulated by phosphorylation/dephosporylation [28]. The kinase involved is the PIKK family member SMG-1 [45,46].

In addition to removing aberrant transcripts, the NMD pathway plays a significant role in the control of gene expression [47]. Microarray analyses indicate that approximately 10% of yeast genes are affected by loss of NMD; most showing increased levels [48-50]. Approximately half of the changes are the result of direct regulation by NMD [50]. Some of the effects on translational readthrough are due to increased expression of the magnesium transporter Alr1p and the elevated cellular magnesium [51]. Other regulatory functions may arise through programmed ribosomal frameshifting, and altered translational start site selection [52-55].

To identify the genetic network in which Tra1 is involved, we selected for second site suppressors that allow growth of strains with the *tra1-L3733A *allele on media containing ethanol. An opal mutation at codon 165 of *upf1 *was identified. Suppression was likely the result of loss of NMD since deletion of *upf1, upf2 *or *upf3 *conferred growth of strains containing *tra1-L3733A*. Suppression was specific for mutations to *TRA1*; phenotypes arising from deletions of SAGA or NuA4 components were not suppressed. Since deletion of *upf1 *only reversed the transcriptional defects of the *tra1 *alleles at a subset of affected promoters, and these same *tra1 *alleles did not affect NMD, we conclude that loss of NMD suppresses the *TRA1 *mutations through crosstalk and/or partial overlap of their regulatory networks.

## Results

**Figure 1:**
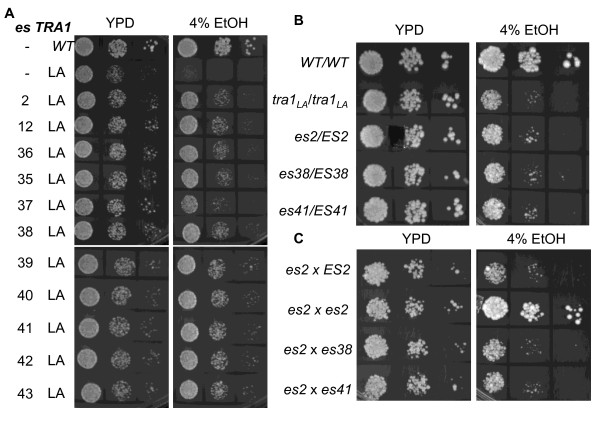
**Extragenic suppression of tra1-L3733A**. A. CY4018 (*tra1-L3733A*, LA) was plated onto YPD media containing 4% ethanol (EtOH) at 30°C. Growing colonies were in succession: colony purified on media containing ethanol, plated on YPD at 30°C, and retested on media containing ethanol. The 11 strains passing this selection (CY5579, *es2*; CY5580, *es12*; CY5581, *es35*; etc.), CY2706 (*TRA1*), and CY4018 (*tra1-L3733A*) were grown to stationary phase, serially diluted and plated onto YPD or YPD containing 4% ethanol at 30°C. **B**. The *es2, es38 *and *es41 *alleles are recessive. Strains CY5579 (*es2 tra1-L3733A*), CY5584 (*es38 tra1-L3733A*) and CY5587 (*es41 tra1-L3733A*) were mated with CY5522 (*tra1-L3733A*) to generate diploid strains homozygous for *tra1-L3733A *and heterozygous for each suppressor. Serial dilutions of these strains as well as the diploid wild-type strain CY5557 and the homozygous *tra1-L3733A *strain CY5558 were spotted onto the indicated plates. **C**. *es2 *forms an independent complementation group. Serial dilutions of diploid strains arising from crosses of haploid strains CY5579 with CY5522 (*es2 *x *ES2*), CY5579 with CY5666 (*es2 *x *es2*), CY5666 with CY5584 (*es2 *x *es38*), and CY5666 with CY5587 (*es2 *x *es41*) were spotted onto the indicated plates.

The FATC domain of Tra1 is critical to the protein's function. Mutation of leucine 3733 to alanine results in temperature sensitivity and slow growth in media containing ethanol, Calcofluor white or rapamycin [23]. These phenotypes provide a tool to probe the genetic network in which *TRA1 *is connected. We selected for extragenic suppressors of the ethanol sensitivity of CY4018, a strain disrupted for the genomic *tra1 *but viable due to *tra1-L3733A *on a *URA3 *centromeric plasmid. Approximately 10^8 ^cells were plated onto YPD plates containing 4% ethanol. Forty-three colonies were isolated for further analysis. Each was retested for the suppression after colony isolation, and then examined for whether the mutation was located on the *tra1-L3733A *containing-plasmid by plasmid shuffling. Eleven strains with extragenic mutations that partially suppressed the slow growth due to *tra1-L3733A *were identified. The growth of these strains on YPD media and YPD containing 4% ethanol is shown in Figure 1a. The strains with the *tra1-L3733A *suppressor alleles (tentatively termed *es2, es12, es35 *etc.) were mated with the MAT***α ****tra1-L3733A *strain CY5522 to determine if the suppressor mutations were dominant or recessive. Figure 1b shows the analysis for CY5579 (*es2*), CY5584 (*es38*), and CY5587 (*es41*). The diploid strains grew slowly under selective conditions, indicating that the suppressor alleles act recessively. Similarly each of the additional *es *alleles was recessive. Complementation groups were analyzed through crosses of the *es *containing strains. As shown in Figure 1c, the diploid crosses of *es2 *with *es38*, and *es41*, grew poorly on media containing 4% ethanol, in contrast to the homozygous diploid containing *es2*. In this way three complementation groups were identified amongst the eleven strains isolated in the screen. One complementation group contained uniquely *es2*; a second group contained uniquely *es35*. The third complementation group contained the remaining nine alleles, including *es38 *and *es41*. A random spore analysis indicated that the ethanol resistance for the *es2, es35 *and *es38 *strains (representing each complementation group) segregated 2:2 suggesting that a single gene was the cause of suppression.

To determine the identity of the suppressor alleles for the *es2 *and *es38*/*es41 *complementation groups, we compared the genome sequence for each of the three strains relative to the parent CY4018. (The third complementation group was not analyzed.) Libraries were prepared and genomic sequencing performed using the ABI SOLiD 4.0 platform at the Centre for Applied Genomics at The Hospital for Sick Children (Toronto, Canada). Approximately 50 million reads were obtained for each sample of which 60% mapped to the reference genome from the *Saccharomyces *Genome Database. Polymorphisms not found in CY4018 or the other complementation group, were analyzed by visual inspection of the sequencing reads. A causative mutation could not be identified within coding or noncoding sequences for the *es38*/*es41 *complementation group. The *es2 *strain contained an opal mutation at tryptophan codon 165 of *UPF1*, truncating the 971-residue protein. To confirm that this allele, now designated *upf1_1-164_*, was responsible for the suppression of *tra1-L3733A*, CY5579 (*upf1_1-164 _tra1-L3733A*) was mated with CY4018 (*tra1-L3733A*), sporulated, and the *UPF1 *alleles in 8 unrelated spore colonies, four exhibiting slow growth and four fast growth, were isolated by PCR and sequenced. The four spore colonies growing slowly on 4% ethanol contained wild-type *UPF1*, whereas the fast growing spore colonies contained *upf1_1-164_*.

**Figure 2:**
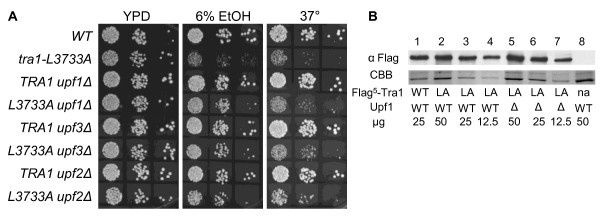
**Deletions of components of the NMD pathway suppress tra1-L3733A**. **A**. Haploid strains CY4353 (*TRA1 UPF1*), CY4103 (*tra1-L3733A UPF1*), CY5932 (*TRA1 upf1Δ*), CY5972 (*tra1-L3733A upf1Δ*), CY5936 (*TRA1 upf3Δ*), CY5983 (*tra1-L3733A upf3Δ*), CY5934 (*TRA1 upf2*Δ), and CY5996 (*tra1-L3733A upf2Δ*) were grown in YPD media to stationary phase, and 10-fold serial dilutions plated onto YPD, YPD containing 6% ethanol, or YPD grown at 37°C. **B**. Expression of Flag^5^-Tra1-L3733A. Yeast strains CY5940 (*Flag^5^-TRA1*; lane 1), CY6004 (*Flag^5^-tra1-L3733A*; lanes 2-4), CY6005 (*Flag^5^-tra1-L3733A upf1Δ0*; lanes 5-7) and BY4741 (*TRA1*, not applicable; lane 8) were grown to stationary phase then diluted 1:20 into YPD and grown for 8 hours at 30°C. Protein extracts were prepared by lysis with glass beads. The indicated amount of protein was separated by SDS-PAGE (5%) and either Western blotted with anti-Flag antibody or stained with Coomassie Brilliant Blue (CBB).

In *S. cerevisiae*, Upf1 is one of three proteins acting in the NMD surveillance complex, the others being Upf2 and Upf3. To determine if loss of this process was responsible for suppression of *tra1-L3733A*, we analyzed the growth of strains deleted for *upf1, upf2 *or *upf3 *in media containing 6% ethanol and at 37°C. As shown in Figure 2A, deletion of any of the components of the NMD surveillance complex partially suppressed the slow growth due to *tra1-L3733A*. Though not documented as a target of NMD, it is possible that loss of NMD suppresses the *tra1-L3733A *allele by increasing the cellular concentration of the protein. We therefore constructed strains that contain an integrated copy of Flag^5^-tagged Tra1 or Tra1-L3733A, and examined expression in the presence or absence of *upf1 *by Western blotting of cell extracts (Figure 2B). The expression of the integrated Flag^5^-Tra1-L3733A is similar to the wild-type protein (compare lanes 1 and 3). Disruption of *upf1 *slightly increased expression of Tra1-L3733A (compare lanes 3 and 6). We estimate this increase to be less than 10%, and suggest that it is not sufficient to account for the suppression.

**Figure 3:**
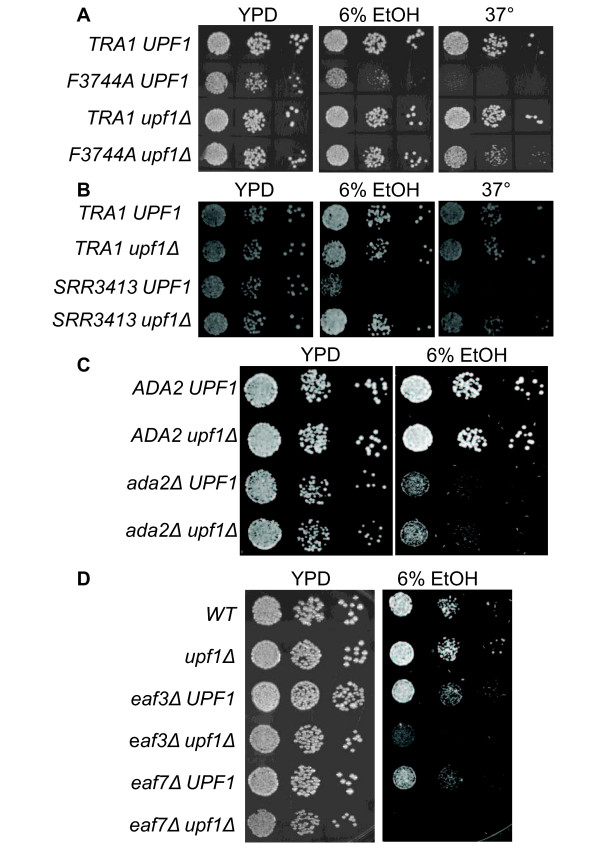
**Suppression by upf1Δ is specific for mutations within the FATC domain of Tra1**. **A**. *upf1Δ *suppression of *tra1*-*F3744A*. Serial dilutions of CY4353 (*TRA1 UPF1*), CY4350 (*tra1-F3744A UPF1*), CY5932 (*TRA1 upf1Δ*), and CY6030 (*tra1-F3744A upf1Δ*) were spotted onto YPD at 30°C or 37°C, or YPD containing 6% ethanol at 30°C. **B**. *upf1Δ *suppression of *tra1-SRR3413*. Serial dilutions of BY4742 (*TRA1 UPF1*), CY5932 (*TRA1 upf1Δ*), CY2200 (*tra1-SRR3413 UPF1*), and CY6111 (*tra1-SRR3413 upf1Δ*) were spotted onto YPD at 30°C or 37°C, or YPD containing 6% ethanol at 30°C. **C**. *upf1Δ *does not suppress *ada2Δ*. Serial dilutions of CY4353 (*ADA2 UPF1*), BY4282 (*ada2Δ UPF1*), CY5932 (*ADA2 upf1Δ*), and CY5979 (*ada2Δ upf1Δ*) haploids were spotted onto the indicated YPD plates. **D**. *upf1Δ *does not suppress *eaf3Δ *or *eaf7Δ*. Serial dilutions of CY4353 (WT), CY5932 (*upf1Δ*), BY7143 (*eaf3Δ UPF1*), CY5980 (*eaf3Δ upf1Δ*), BY2940 (*eaf7Δ UPF1*), and CY5976 (*eaf7Δ upf1Δ*) haploids were spotted onto YPD at 30°C or YPD containing 6% at 35°C.

We next addressed the allele specificity of the suppression. Alteration of the terminal phenylalanine of tra1 to alanine results in slow growth in media containing ethanol. A strain deleted for *upf1 *and containing *tra1-F3744A *was constructed and its growth compared to the single mutant strains (Figure 3a). Deletion of *upf1 *suppressed the slow growth due to *tra1-F3744A*, at least to the same extent as for *tra1-L3733A*. Deletion of *upf1 *also suppressed slow growth at 37°C and on media containing 6% ethanol caused by *tra1*-*SRR3413*, a triple alanine scanning mutation to residues 3,413-3,415 within the PI3K domain ([17]; Figure 3b). Deletions of other components of the SAGA and NuA4 complexes were then examined (Figure 3c and 3d). Deletion of *ada2 *results in slow growth on media containing ethanol; however, unlike the *tra1 *mutations, the slow growth caused by *ada2Δ *was not suppressed by *upf1Δ *(Figure 3c). Disrupting the NuA4 components Eaf3 or Eaf7 results in slow growth at 35°C in media containing 6% ethanol. Deletion of *upf1 *did not suppress this phenotype for either the *eaf3Δ *or *eaf7Δ *strains (Figure 3d). In fact synthetic slow growth was observed on plates containing 6% ethanol for the double mutant strains. This latter result agrees with the synthetic slow growth reported for mutations of *esa1 *and *eaf7 *with *upf1 *and *upf3*, respectively [56,57].

**Figure 4:**
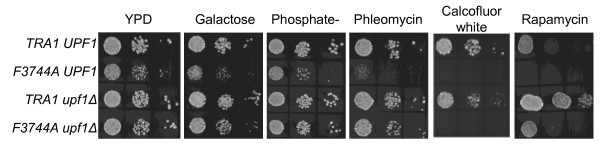
**Phenotypes of TRA1-F3744A suppressed by upf1Δ**. Serial dilutions of yeast strains CY4353 (*TRA1 UPF1*), CY4350 (*tra1-F3744A UPF1*), CY5932 (*TRA1 upf1Δ*), and CY6030 (*tra1-F3744A upf1Δ*) haploids were spotted onto YPD, YP plus 2% galactose, YPD depleted of phosphate, or YPD containing 1 μg/mL phleomycin, 10 μg/mL Calcofluor white, or 2 nM rapamycin.

Alterations to the FATC domain cause slow growth in a number of conditions [23]. We analyzed which of these in addition to the suppression of ethanol and temperature sensitivity are suppressed by *upf1Δ*. As shown in Figure 4, deletion of *upf1 *suppressed the slow growth resulting from *tra1-F3744A *when galactose is the carbon source, when phosphate is depleted, and when phleomycin (a DNA damaging agent) is present in the media. In contrast deletion of *upf1 *did not suppress slow growth due to *tra1-F3744A *in the presence of the cell wall destabilizing agent Calcofluor white, and only had a modest effect with rapamycin. The latter was particularly interestingly, given that deletion of *upf1 *in the wild-type *TRA1 *background decreased sensitivity to rapamycin (compare *TRA1 UPF1 *with *TRA1 upf1Δ*).

**Figure 5:**
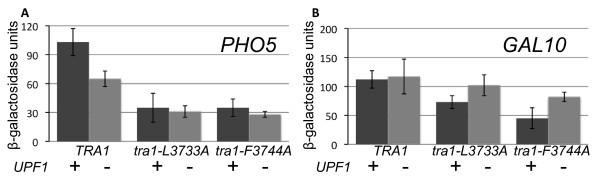
**Expression of PHO5-lacZ and GAL10-lacZ in upf1Δ strains**. **A**. Yeast strains CY4353 (*TRA1 UPF1*), CY5932 (*TRA1 upf1Δ*), CY4103 (*tra1-L3733A UPF1*), CY5972 (*tra1-L3733A upf1Δ*), CY4350 (*tra1*-F3744A *UPF1*), and CY6030 (*tra1*-F3744A *upf1Δ*) were transformed with a *LEU2 *centromeric plasmid containing a *PHO5-lacZ *fusion, grown to stationary phase in media depleted of leucine, washed three-times with water, diluted into YPD media depleted of phosphate, grown for 16 hr at 30°C and β-galactosidase activity determined, normalizing to cell density. **B**. *GAL10-lacZ *was analyzed as above with initial cultures grown in raffinose containing media and shifting to 2% galactose. The P values for the statistical significance of the difference of *UPF1 *versus *upf1Δ *for *TRA1-L3733A *and *tra1-F3744A *are 0.006 and 0.001 (n = 6), respectively.

We next addressed whether disruption of *upf1 *would reverse the transcriptional defects caused by the *TRA1 *mutations. Expression of two *lacZ *promoter fusions was examined: *PHO5-lacZ *and *GAL10-lacZ. tral-L3733A *and *tra1-F3744A *decreased activated expression of *PHO5-lacZ *to approximately one-third of the wild-type level (Figure 5a). Interestingly, the strain containing a disruption of *upf1 *in the context of wild-type *TRA1 *also showed decreased expression (approximately 2-fold). Furthermore, disruption of *upf1 *in the context of either tral-L3733A or *tra1*-F3744A did not restore transcription. As shown in Figure 5b expression of *GAL10-lacZ *was also reduced by *tral-L3733A *and *tra1-F3744A*, but in contrast to *PHO5-lacZ *not by deletion of *upf1*. In addition, at *GAL10 *the transcriptional defect due to the *tral *alleles was partially reversed by *upf1Δ*. Together these results suggest that in some, but not all, cases disruption of *upf1 *may suppress *tra1*-induced phenotypes by regulating transcription (most likely indirectly) of common genes.

**Figure 6:**
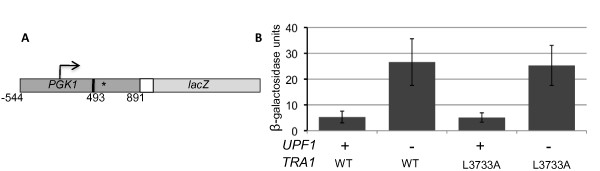
**Expression of frameshifted PGK1 in tra1-L3733A and tra1-F3744A strains**. **A**. *PGK1_fs_-lacZ*. The promoter and coding reading of *PGK1 *from-544 to 891 was synthesized by PCR, and the reading frame changed after codon 166 by endfilling of the *Asp*718 site (493 bp, solid line) and religation. This results in a stop 26 codons 3' to the frameshift (asterick). *PGK1 *is fused to residues 305-403 of *HIS3 *(white box) followed by the coding sequence of *lacZ*. B. Yeast strains CY4353 (*TRA1 UPF1*), CY5932 (*TRA1 upf1Δ*), CY4103 (*tra1-L3733A UPF1*), and *CY5972 *(*tra1-L3733A upf1Δ*) were transformed with a *LEU2 *centromeric plasmid containing *PGK1_fs_-lacZ*. Cultures were grown to stationary phase in media depleted of leucine, inoculated at a 100-fold dilution in YPD media, grown for 16 hr at 30°C and β-galactosidase activity determined, normalizing to cell density.

Our previous studies with *TRA1 *have linked phenotypes resulting from its mutation with transcriptional change [17,23]. The observation that disrupting *upf1 *did not restore transcription in all cases, suggested two models for how loss of NMD might suppress the FATC domain mutations. The first predicts a direct link between NMD and Tra1. If Tra1 is a negative regulator of NMD, Tra1-L3733A might increase NMD, decreasing the level of certain mRNA transcripts, which in turn could cause growth-related phenotypes. Loss of the NMD pathway would reverse the effect. The interaction between SAGA component Sgf29 and Upf1 [58], and the phosphorylation of Upf1 by the PIKK member SMG-1 in metazoans [45,46,59], are consistent with the possibility of direct regulation. The second model predicts a less specific interaction between Tra1 and NMD. The *tra1 *mutations through its action in SAGA, NuA4 or independently [13] alter expression of a set of genes. Some of these genes, or genes with epistatic relationships, may be regulated through mRNA turnover and/or translational readthrough by processes involving NMD. This possibility is enhanced by the scope of both networks and their generally reciprocal nature. By eliminating NMD the expression of genes that directly or indirectly intersect in the pathways may return to near normal, effectively compensating for diminished Tra1 activity. To differentiate between these models, we analyzed whether *tral-L3733A *alters NMD. We constructed a *PGK1-lacZ *fusion (*PGK1_fs_-lacZ*; Figure 6a) frameshifted at codon 166 and including those sequences required for NMD, -544 to 891 [34,60]. Previous mRNAseq experiments have indicated that *PGK1 *expression in YPD media is relatively unaffected by mutations within the FATC domain of Tra1 [23]. Deletion of *upf1 *results in a 5-fold increase in expression of β-galactosidase from the *PGK1_fs_-lacZ *fusion in comparison to a wild-type *UPF1 *strain (Figure 6b), verifying the use of this construct to monitor NMD. Indicative of Tra1 not having a direct role in NMD, expression of *PGK1_fs_-lacZ *was unaffected by tral-L3733A. In light of this result and the broad role for NMD in gene expression, we thus favor a model whereby deletion of NMD suppresses the *tra1 *alleles through its reciprocal action on an overlapping set of genes.

## Discussion

We have identified a genetic interaction between components of the nonsense mediated decay pathway and *tra1*, through random selection for mutations that suppress the ethanol sensitivity of a *tra1-L3733A *strain. Our initial selection identified a nonsense mutation at codon 165 of *UPF1*. Similar suppression was found with deletions of *upf1, upf2 *and *upf3 *indicating that loss of nonsense mediated decay was the likely cause. Our study demonstrates a relationship between the Tra1 and NMD regulatory networks, and further emphasizes the general importance of NMD in gene expression.

The suppression mediated by deletion of *upf1 *was specific for certain phenotypes arising from *tra1 *mutations. Slow growth due to high temperature, ethanol, low phosphate, galactose as the primary carbon source, and phleomycin was suppressed. Slow growth due to rapamycin or Calcofluor white was not. The suppression mediated by *upf1Δ *was also specific for mutations within *TRA1*, not suppressing deletions of the SAGA component Ada2 or the NuA4 components Eaf3 or Eaf7. In the case of the latter two disruptions, synthetic slow growth was observed.

We propose the suppression of *tra1-L3733A *and *tra1*-*F3744A *caused by loss of NMD is the result of crosstalk and/or direct overlap in the networks regulated by these genes. In this model, altered (most often decreased) expression resulting from the *tra1 *mutations would be partially reversed by reducing mRNA turnover (or enhancing translational readthrough) in the NMD deficient backgrounds. Also along this line, a gene regulated by NMD may encode a protein that directly or indirectly regulates some of the functions of Tra1. Our reasons for preferring this indirect mechanism for suppression are the following. First, consistent with this model nonsense mediated decay has a broad role in gene regulation. Some of NMD's roles relate to the removal of aberrant transcripts that have acquired nonsense codons. Other roles relate to control mechanisms that utilize the pathway to remove mRNAs that would otherwise be functional. As such NMD influences approximately 10% of yeast genes [48,49]--likely an underestimate of the extent of its control as this is in rich media. Clearly the breadth of the NMD effect indicates that NMD is of global importance in yeast regulatory pathways, not only affecting aberrant transcripts. The majority of genes are upregulated in response to loss of NMD; this contrasts to the generally more prevalent decreased expression observed upon mutation of *tra1 *[17,23]. Thus the prevalence of genes affected and reciprocal nature of loss of NMD and Tra1 could result in their neutralization in a double mutant background. Indeed, it is the scope of NMD that likely explains why its loss can also suppress mutations of other globally important yet functionally diverse factors required for gene expression (for example: *TAF6, TAF9 *and *RAP1 *[57], *PAF1 *[61], and *BRE1 *[62]).

This indirect model for suppression accommodates the finding that only a subset of *tra1-L3733A *phenotypes is suppressed by loss of NMD. Suppression would require that Tra1 and NMD regulate key genes responsible for the phenotype (also see below). Similarly, specificity for *TRA1 *mutations may be accounted for if other NuA4 and/or SAGA components influence genes to an extent that is not sufficiently reversed by NMD. Finally, the finding that loss of NMD affects promoter-dependent events of some *tra1*-effected genes (for example *GAL10*) but not others (for example, *PHO5*) is more consistent with an indirect mechanism for suppression that could act through distinct genes and/or steps in gene expression, rather than by restoring Tra1 function.

Alternative models for suppression by *upf1Δ *are possible if Tra1 were involved in NMD. The finding that *tra1-L3733A *and *tra1*-*F3744A *did not alter expression of an internally frame-shifted *PGK1 *reporter plasmid, suggests that Tra1 is not directly involved in NMD. The lack of direct relationship between Tra1 and NMD is consistent with Tra1 acting in the nucleus, whereas in *S. cerevisiae *NMD is primarily a cytoplasmic process regulating post-transcriptional events [63].

We have not pinpointed the genes whose regulation by NMD allows suppression of the phenotypes caused by *tra1-L3733A*. Because of epistatic relationships, the key genes affected by *tra1-L3733A *and NMD may not be identical. It is also possible that small changes in multiple target genes could cause suppression; this would make identification of relevant targets difficult. Expression screening of mRNAs to detect changes in profiles may be complicated because the genes are likely stress induced, and will differ from condition to condition. Moreover, some aspects of NMD relate specifically to translation, and will not be seen by RNA profiling. Nonetheless, we have compared gene expression profiles in YPD media for *tra1-L3733A *[23] and *upf1Δ *[49] strains. Of the 79 genes with reduced expression due to *tra1-L3733A *(twofold or greater), six display increased expression of twofold or greater with *upf1Δ *(one has decreased expression). This ratio, given that it approximates the 10% of the genome regulated by NMD, does not support a specific overlap in the pathways, however it does emphasize the generally reciprocal nature of Tra1 and NMD. The six genes with reciprocal changes in expression are YBL107C, YER187W, *MIP6 *(YHR015W), *JMN1 *(YMR294W), YNR071C and YOL014W. Of these only *MIP6 *and *JMN1 *have characterized functions. *MIP6 *is of potential relevance since it encodes a protein with putative RNA binding motifs, and was identified in a two-hybrid analysis as interacting with the Mex67, an mRNA export factor [64].

When expressed on a centromeric plasmid from the *DED1 *promoter, Tra1-L3733A is less abundant than the wild type protein [23]. We do not observe this decrease when FATC mutations are integrated into the genome. For this reason the functional experiments performed in this analysis were with genomically encoded *tra1-L3733A *and *tra1-F3744A*. Based on the recent results of Stirling et al. [65], who show a link between chromosome instability and components of the ASTRA complex, we believe that the plasmid versions may be less well expressed due to decreased stability of the plasmid for the mutant versions of *tra1*.

In a recent independent selection for suppressors of *tra1-F3744A*, we identified two alleles of the ASTRA component Tti2 (Genereaux et al. Genetics, in press). The tti2 alleles also suppress *tra1-L3733A*, but not deletions of components of SAGA or NuA4 components. The tti2 alleles, unlike *upf1_1-164_*, acted dominantly. This as well as complementation experiments with *TTI2 *(not shown) suggests that *es38*/*41 *and *es35 *represent additional independent suppressor alleles.

## Conclusion

We have demonstrated a genetic interaction between *TRA1 *and genes of the nonsense mediated decay pathway. In a recessive manner, deletion of *upf1 *partially suppressed the growth related defects of *tra1-L3733A, tra1*-*F3744A *and *tra1*-*SRR3413*, mutations within the FATC and PI3K domains. The suppression was specific for *TRA1 *mutations; no effect was seen for deletions of other SAGA or NuA4 components. A subset of phenotypes attributable to the FATC mutations was suppressed; furthermore, not all transcriptional defects were reversed by deletion of *upf1*. We suggest that the suppression relates to the breadth and overlapping, yet generally reciprocal nature of the gene regulatory pathways in which Tra1 and the NMD components are involved.

## Methods

### Yeast strains and growth

Strains for selection of suppressor mutations are derivatives of KY320 ([66]; see Table 1) and the isogenic MAT**α **strain CY4413. CY1021 contains a genomic disruption of *tra1 *and is maintained by a plasmid copy of myc-tagged *TRA1 *expressed from the *DED1 *promoter [2]. CY3003 [23] and CY4018 were obtained from CY1021 by plasmid shuffling and contain myc^9^-tagged *tra1-L3733A *expressed from the *DED1 *promoter on YCplac22 [67] or a *URA3 *centromeric plasmid (YCplac22u) derived from YCplac22 by switching *TRP1 *to *URA3*, respectively [68]. CY5522 is the MAT**α **equivalent of CY4018 and was generated by mating CY4413 with CY4018. After sporulation, a *MAT**α ***spore colony was isolated that required the plasmid copy of *tra1-L3733A *for growth. Strains carrying *tra1-L3733A *and extragenic suppressors (hereafter defined as es alleles) *es2 *(CY5579), *es12 *(CY5580), *es35 *(CY5581), *es36 *(CY5582), *es37 *(CY5583), *es38 *(CY5584), *es39 *(CY5585), *es40 *(CY5586), *es41 *(CY5587), *es42 *(CY5588), and *es43 *(CY5750) were derived from CY4018 using the selection scheme described below. CY5666 (*es2*), CY5758 (*es38*), and CY5603 (*es41*) are *MAT**α ***equivalents of CY5579, CY5584, and CY5587, respectively, and were made after mating with CY5522. *MAT**α ***spore colonies carrying the suppressor were selected based on their ability to grow at high temperature and on plates containing 4% ethanol.

**Table 1:** Yeast strains

Strain number	Description	*TRA1 *plasmid	Reference
CY1021	Isogenic to KY320 except *tra1Δ*	YCplac22-*myc-TRA1*	[2]
CY2706	Isogenic to CY1021	YCplac22-*myc_9_-TRA1*	[23]
CY3003	Isogenic to CY1021	*YCplac22-myc_9_-tra1-L3733A*	[23]
CY4018	Isogenic to CY1021	*YCplac22u-myc_9_*-*tra1*-*L3733A*	This study
CY5522	Isogenic to CY3003 except *MATα*	*YCplac22-myc_9_-tra1-L3733A*	This study
CY5557	Diploid cross of CY2706 and CY4413	*YCplac22-myc_9_-TRA1*	This study
CY5558	Diploid cross of CY4018 and CY5522	*YCplac22-myc_9_-tra1-L3733A*	This study
CY5579	isogenic to CY4018 except *es2 *(*upf1_1-164_*)	*YCplac22u-myc_9_-tra1-L3733A*	This study
CY5580	isogenic to CY4018 except *es12*	*YCplac22u myc_9_-tra1- L3733A*	This study
CY5581	isogenic to CY4018 except *es35*	*YCplac22u myc_9_-tra1- L3733A*	This study
CY5582	isogenic to CY4018 except *es36*	*YCplac22u myc_9_-tra1- L3733A*	This study
CY5583	isogenic to CY4018 except *es37*	*YCplac22u myc_9_-tra1- L3733A*	This study
CY5584	isogenic to CY4018 except *es38*	*YCplac22u myc_9_-tra1- L3733A*	This study
CY5585	isogenic to CY4018 except *es39*	*YCplac22u-myc_9_*-*tra1*-*L3733A*	This study
CY5586	isogenic to CY4018 except *es40*	*YCplac22u-myc_9_*-*tra1*-*L3733A*	This study
CY5587	isogenic to CY4018 except *es41*	*YCplac22u-myc_9_-tra1- L3733A*	This study
CY5588	isogenic to CY4018 except *es42*	*YCplac22u-myc_9_*-*tra1*-*L3733A*	This study
CY5750	isogenic to CY4018 except *es43*	*YCplac22u-myc_9_-tra1-L3733A*	This study
CY5666	Isogenic to CY5522 except *es2 *(*upf1*_1-164_)	*YCplac22-myc_9_-tra1-L3733A*	This study
CY5758	Isogenic to CY5522 except *es38*	*YCplac22-myc_9_-tra1-L3733A*	This study
CY5603	Isogenic to CY5522 except *es41*	*YCplac22-myc_9_-tra1-L3733A*	This study
CY5688	Isogenic to CY2706 except *es2 *(*upf1*_1-164_)	*YCplac22u-myc_9_-TRA1*	This study
CY5690	Isogenic to CY2706 except *es38*	*YCplac22u-myc_9_-TRA1*	This study
CY5691	Isogenic to CY2706 except *es41*	*YCplac22u-myc_9_-TRA1*	This study
KY320	*MATa ura3-52 ade2-101 trp1-Δ1 lys2-801 his3-Δ200 leu2::PET56*		[66]
CY4413	*MATα ura3-52 ade2-101 trp1-Δ1 lys2-801 his3-Δ200 leu2::PET56*		
CY2222	*MATa can1Δ::STE2pr-SpHIS5 lyp1Δ his3Δ 1 leu2Δ0 ura3Δ0 met10 LYS2+ TRA1-SRR3413-URA3*		[69]
BY4741	*MATa his3Δ1 leu2Δ0 met15Δ0 ura3Δ0*		[70]
BY4742	*MATα his3Δ1 leu2Δ0 lys2Δ0 ura3Δ0*		[70]
BY2940	*MATa his3Δ1 leu2Δ0 met15Δ0 ura3Δ0 eaf7::KanMX*		[70]
BY4282	*MATa his3Δ1 leu2Δ0 met15Δ0 ura3Δ0 ada2::KanMX*		[70]
BY7143	*MATa his3Δ1 leu2Δ0 met15Δ0 ura3Δ0 eaf3::KanMX*		[70]
BY41905	*MATa/α his3Δ1/his3Δ1 leu2Δ0 lys2Δ0/LYS2 MET15/met15Δ0 ura3Δ0/ura3Δ0 + can1Δ::LEU2 + -MFA1pr-HIS3/CAN1+ upf2Δ::KanMX*		[71]
BY44702	*MATa/α his3Δ1/his3Δ1 leu2Δ0 lys2Δ0/LYS2 MET15/met15Δ0 ura3Δ0/ura3Δ0 can1Δ::LEU2 + -MFA1pr-HIS3/CAN1+ upf3Δ::KanMX*		[71]
BY46214	*MATa/α his3Δ1/his3Δ1 leu2Δ0 lys2Δ0/LYS2 MET15/met15Δ0 ura3Δ0/ura3Δ0 can1Δ::LEU2 + -MFA1pr-HIS3/CAN1+ upf1Δ::KanMX*		[71]
CY4350	*MATa ura3Δ0 his3Δ0 leu2Δ0 TRA1-F3744A-HIS3*		[23]
CY4353	*MATa ura3Δ0 his3Δ0 leu2Δ0 TRA1-HIS3*		[23]
CY4103	*MATa ura3Δ0 his3Δ0 leu2Δ0 TRA1-L3733A-HIS3*		[23]
CY5932	*MATa his3Δ1 leu2Δ0 ura3Δ0 upf1Δ::KanMX*		This study
CY5934	*MATa his3Δ1 leu2Δ0 ura3Δ0 upf2Δ::KanMX*		This study
CY5936	*MATa his3Δ1 leu2Δ0 ura3Δ0 upf3Δ::KanMX*		This study
CY5937	*MATα his3Δ1 leu2Δ0 ura3Δ0 upf3Δ::KanMX*		This study
CY5938	*MATa his3Δ1 leu2Δ0 ura3Δ0 upf1Δ::nat1*		This study
CY5939	*MATα his3Δ1 leu2Δ0 ura3Δ0 upf1Δ::nat1*		This study
CY5940	*MATα ura3Δ0 his3Δ0 leu2Δ0 URA3-Flag^5^-TRA1*		This study
CY5967	*MATα ura3Δ0 his3Δ0 leu2Δ0 tra1-L3733A-HIS3*		This study
CY5968	*MATα his3Δ1 leu2Δ0 ura3Δ0 upf1Δ::KanMX*		This study
CY5972	*MATa ura3Δ0 his3Δ0 leu2Δ0 tra1-L3733A-HIS3 upf1Δ::KanMX*		This study
CY5976	*MATα his3Δ1 leu2Δ0 met15Δ0 ura3Δ0 eaf7::KanMX upf1Δ::nat1*		This study
CY5979	*MATα his3Δ1 leu2Δ0 met15Δ0 ura3Δ0 ada2::KanMX upf1Δ::nat1*		This study
CY5980	*MATα his3Δ1 leu2Δ0 met15Δ0 ura3Δ0 eaf3::KanMX upf1Δ::nat1*		This study
CY5983	*MATa ura3Δ0 his3Δ0 leu2Δ0 tra1-L3733A-HIS3 upf3Δ::KanMX*		This study
CY5996	*MATa ura3Δ0 his3Δ0 leu2Δ0 tra1-L3733A-HIS3 upf2Δ::KanMX*		This study
CY6004	*MATa ura3Δ0 his3Δ0 leu2Δ0 URA3-Flag5-tra1-L3733A-HIS3*		This study
CY6005	*MATa ura3Δ0 his3Δ0 leu2Δ0 URA3-Flag5-tra1-L3733A-HIS3 upf1Δ::KanMX*		This study
CY6030	*MATa ura3Δ0 his3Δ0 leu2Δ0 tra1-F3744A-HIS3 upf1Δ::KanMX*		This study
CY6102	*MATα ura3Δ0 leu2Δ0 tra1-SRR3413-URA3 upf1Δ::KanMX*		This study

Yeast strains deleted for *upf1 *(CY5932), *upf3 *(CY5936) and *upf2 *(CY5934) were derived from the diploid strains, BY46214, BY44702 and BY41905 [71], respectively, by selecting Kan^r ^spore colonies. These *MAT**a ***strains were mated with CY4057 (*tra1-L3733A*; [23]), to yield after sporulation and selection of Kan^r ^His ^- ^spore colonies CY5972 (*upf1Δ tra1-L3733A*), CY5983 (*upf3Δ tra1-L3733A*), and CY5996 (*upf2Δ tra1-L3733A*). Similarly, CY6030 (*upf1Δ tra1*-*F3744A*) was generated after mating of CY4351 (*tra1*-F3744A) and CY5932. CY5939 (*upf1Δ*::nat1) was obtained by gene replacement of *Kan^r ^*with *nat1*. Double mutant strains of *upf1Δ*::*nat1 *with *ada2Δ *(CY5979), *eaf3Δ *(CY5980), and *eaf7Δ *(CY5976) were obtained after mating and sporulation of the diploids of crosses of CY5939 with BY4282, BY7143, and BY2940, respectively. CY6111 (*tra1-SRR3413 upf1Δ*) was derived after mating of CY2220 [69] and CY5932, and selecting Ura + Kan^r ^spore colonies. CY6004, CY6005 contain a *TRA1 *allele with a N-terminal Flag^5^-tag integrated with a *URA3 *marker.

Growth comparisons were performed on YP media containing 2% glucose (YPD) selective plates after 3-5 days at 30°C unless stated otherwise. Standard concentrations used for the selections are as follows: 7.5 μg/ml Calcofluor White (Sigma-Aldrich, Inc.), 4% or 6% ethanol, 1.0 μg/ml phleomycin (Sigma-Aldrich, Inc.), and 2 nM rapamycin (LC Laboratories, Woburn Ma). We note that the KY320 background is more sensitive to ethanol than the BY4741 background, accounting for the use of either 4% or 6% ethanol, respectively.

### DNA molecules

Myc-tagged *tra1 *alleles on the centromeric *TRP1 *(YCplac22) or *URA3 *(YCplac33) plasmids have been described [17,23]. *PHO5 *(-452 to +47) and *GAL10 *(-595 to -245) promoter-*lacZ *fusions in the *LEU2 *centromeric plasmid YCp87 have been described [17,72]. The *PGK1-lacZ *fusion with a frameshift mutation (*PGK1fs-lacZ*) was constructed by synthesizing the gene from -544 to +891 by PCR using oligonucleotides 5'-GCGGATCCACGTGGCCTCTTATCGAG-3' and 5'-CTCAAGCTTCCTTGGTGTTGGCATCAGCAGAG-3', digesting the gene with *Asp*718, creating blunt ends with the Klenow fragment of DNA polymerase and religation.

### Isolation of suppressor strains

Six cultures of CY4018 were grown overnight in YPD. Approximately 100 million cells were plated onto 200 YPD plates containing 4% ethanol and incubated at 30°C for 5 days. Fast growing colonies were colony purified and retested. *YCplac22-tra1-L3733A *was transformed into each potential suppressor strain and *YCplac22u-myc_9_-tra1-L3733A *shuffled out on 5-fluoroorotic acid. Strains that retained their ability to grow on 4% ethanol were defined as containing extragenic suppressors.

### Genomic sequence analysis

Genomic DNA was prepared from10 mL of lyticase treated cells [73]. Approximately 5 μg of DNA from each sample was sent to the Centre for Applied Genomics (Toronto, Ontario). DNA library construction and next-generation sequencing using paired-end reads was performed at the Centre. Samples were sequenced using the Applied Biosystems SOLiD 4.0 next-generation sequencing platform. The sequencing was performed in a single lane with multiplexing that included 11 additional unrelated samples. The *Saccharomyces cerevisiae *genome sequence was downloaded from the *Saccharomyces *Genome Database (SGD; [74]) on March 24, 2011. Custom Shell and Perl scripts were written for the sequencing analysis. The program Bowtie [75], allowing up to three mismatches per read, was used to map the colorspace reads to each chromosome of the yeast genome and obtain mapped reads in SAM format (Sequence Alignment/Map; [76]). The VCF (variant call format) from SAMtools [76] was used to obtain a raw list of polymorphisms from the mapped reads. Those reads with a Phred quality score below 20 were eliminated to obtain a filtered list of polymorphisms. A custom Perl script was written to eliminate the background polymorphisms found in wild-type samples.

### β-galactosidase assays

Yeast strains containing *lacZ*-promoter fusions were grown to stationary phase in media lacking leucine. Assays with *PHO5-lacZ *in media depleted of phosphate and *GAL10-lacZ *in media containing galactose as the sole carbon source were performed as described in Mutiu et al. [17] and Brandl et al. [72], respectively, with ο-nitrophenol-β-D-galactosidase as substrate and normalizing values to cell density. Assay of *PGK1_fs_-lacZ *was performed similarly in YPD media. Results presented are from a minimum of four replicates with the standard errors indicated.

### Western blotting

Western blotting was performed using PVDF membranes and anti-Flag (M2; Sigma-Aldrich) antibody as described previously [17].

## Authors' contributions

SK and CJB performed the experiments and co-wrote the manuscript. GBG and SK performed the bioinformatics analysis. All authors have read and approved the manuscript.
